# Loss-of-function variants in myocardin cause congenital megabladder in humans and mice

**DOI:** 10.1172/JCI128545

**Published:** 2019-11-04

**Authors:** Arjan C. Houweling, Glenda M. Beaman, Alex V. Postma, T. Blair Gainous, Klaske D. Lichtenbelt, Francesco Brancati, Filipa M. Lopes, Ingeborg van der Made, Abeltje M. Polstra, Michael L. Robinson, Kevin D. Wright, Jamie M. Ellingford, Ashley R. Jackson, Eline Overwater, Rita Genesio, Silvio Romano, Letizia Camerota, Emanuela D’Angelo, Elizabeth J. Meijers-Heijboer, Vincent M. Christoffels, Kirk M. McHugh, Brian L. Black, William G. Newman, Adrian S. Woolf, Esther E. Creemers

**Affiliations:** 1 Department of Clinical Genetics, Amsterdam UMC, Amsterdam, Netherlands.; 2 School of Biological Sciences, Faculty of Biology, Medicine and Health, University of Manchester, Manchester, United Kingdom.; 3 Manchester Centre for Genomic Medicine and Royal Manchester Children’s Hospital, Manchester University NHS Foundation Trust, Manchester Academic Health Science Centre, Manchester, United Kingdom.; 4 Department of Medical Biology, Amsterdam UMC, Amsterdam, Netherlands.; 5 Cardiovascular Research Institute, UCSF, San Francisco, California, USA.; 6 Department of Medical Genetics, University Medical Center Utrecht, Utrecht, Netherlands.; 7 Laboratory of Molecular and Cell Biology, Istituto Dermopatico dell’Immacolata, IDI-IRCCS, Rome, Italy.; 8 Department of Life, Health and Environmental Sciences, University of L’Aquila, Aquila, Italy.; 9 Department of Experimental Cardiology, Amsterdam UMC, Amsterdam, Netherlands.; 10 Department of Biology, Miami University, Oxford, Ohio, USA.; 11 Center for Clinical and Translational Research, The Research Institute, Nationwide Children’s Hospital, Columbus, Ohio, USA.; 12 Department of Molecular Medicine and Medical Biotechnology, University of Naples Federico II, Naples, Italy.

**Keywords:** Muscle Biology, Genetic diseases, Molecular genetics, Urology

## Abstract

Myocardin (MYOCD) is the founding member of a class of transcriptional coactivators that bind the serum-response factor to activate gene expression programs critical in smooth muscle (SM) and cardiac muscle development. Insights into the molecular functions of MYOCD have been obtained from cell culture studies, and to date, knowledge about in vivo roles of MYOCD comes exclusively from experimental animals. Here, we defined an often lethal congenital human disease associated with inheritance of pathogenic *MYOCD* variants. This disease manifested as a massively dilated urinary bladder, or megabladder, with disrupted SM in its wall. We provided evidence that monoallelic loss-of-function variants in *MYOCD* caused congenital megabladder in males only, whereas biallelic variants were associated with disease in both sexes, with a phenotype additionally involving the cardiovascular system. These results were supported by cosegregation of *MYOCD* variants with the phenotype in 4 unrelated families by in vitro transactivation studies in which pathogenic variants resulted in abrogated SM gene expression and by the finding of megabladder in 2 distinct mouse models with reduced *Myocd* activity. In conclusion, we have demonstrated that variants in *MYOCD* result in human disease, and the collective findings highlight a vital role for MYOCD in mammalian organogenesis.

## Introduction

Urinary tract and kidney malformations often result in termination of pregnancy after being detected on ultrasound screening, and these anomalies are also a major cause of renal failure in surviving children (1). While the genetic bases of kidney malformations are well-recognized (2), with pathogenic variants reported in transcription and growth factors that drive metanephric differentiation, the possible genetic causes of congenital ureter and bladder anomalies are much less clear (3). A striking urinary tract phenotype is megabladder, with a first trimester prevalence of 1:330–1670 (4). Megabladder accompanied by a thinned and wrinkled abdominal wall overlying the bladder is called prune belly syndrome (PBS). Megabladder and PBS are accompanied by kidney glomerular cysts considered to be secondary to fetal urinary flow obstruction (5). Some megabladders are associated with anatomically obstructed bladder outflow tracts, and these bladders have increased smooth muscle (SM) in their walls. Other megabladders are examples of functional outflow obstruction and have patent urethras and thin bladder walls (6). We hypothesized that mutations in genes affecting SM differentiation can cause megabladder and PBS.

Myocardin (MYOCD) is the founding member of a class of powerful transcriptional coactivators that bind to serum response factor (SRF) to activate cardiac- and SM-specific gene expression programs (7, 8). Complete loss of Myocd in mice causes embryonic lethality at midgestation due to failure of SM cell differentiation, whereas heterozygous knockout mice appear normal (9). Conditional mouse models subsequently defined the function of Myocd in postnatal development. Specifically, genetic ablation of *Myocd* in adult hearts causes heart failure due to loss of sarcomere structure and increased cardiomyocyte apoptosis (10). Genetic deletion of *Myocd* specifically in SM revealed that Myocd maintains vascular and visceral SM homeostasis postnatally (11). Despite these unique and important functions of Myocd in mice, no human genetic disorder associated with *MYOCD* variants has yet been defined. Here, we describe *MYOCD* loss-of-function variants in 9 individuals from 4 families with the megabladder/PBS spectrum, and we support these observations on urinary tract maldevelopment with *Myocd* mutant mouse models.

## Results and Discussion

**Figure 1:**
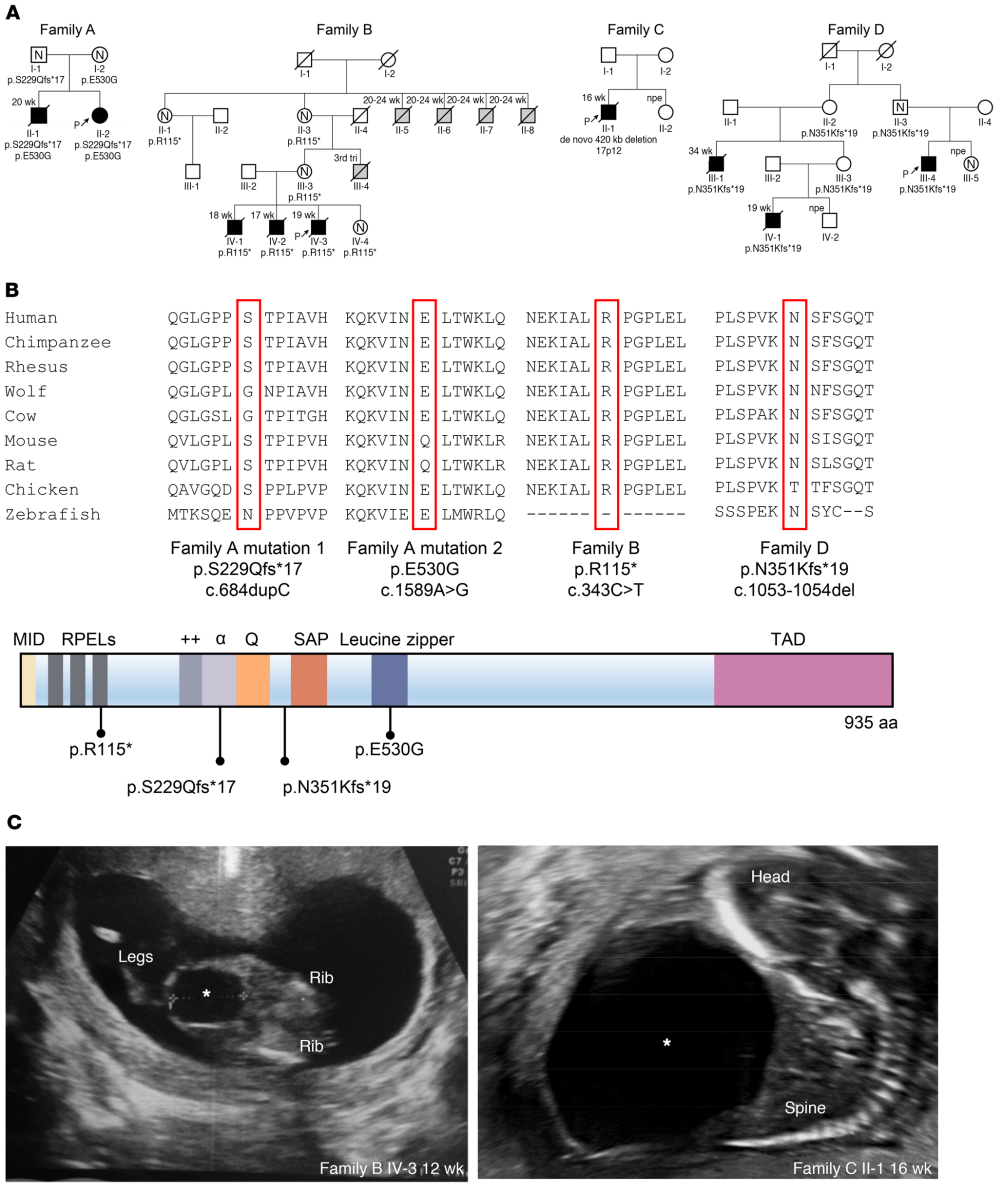
Identification of *MYOCD* variants in 4 families with congenital megabladder. (**A**) Pedigrees of 4 families presenting with congenital megabladder. Affected individuals are marked with black symbols. Available genotypes are shown beneath symbols. Slashed symbols denote deceased individuals. Gestational age is indicated above the symbol. Gray symbols denote stillbirths with external features consistent with PBS. N, normal bladder ultrasound; P with arrow, proband of the family; npe, normal prenatal echo. (**B**) Schematic diagram showing functional domains within MYOCD and location of the identified mutations (7). Conservation of respective amino acid positions with the mutated residues are highlighted. (**C**) Ultrasound images showing enlarged bladder of indicated fetuses of families B and C; asterisks denote bladder.

**Figure 2:**
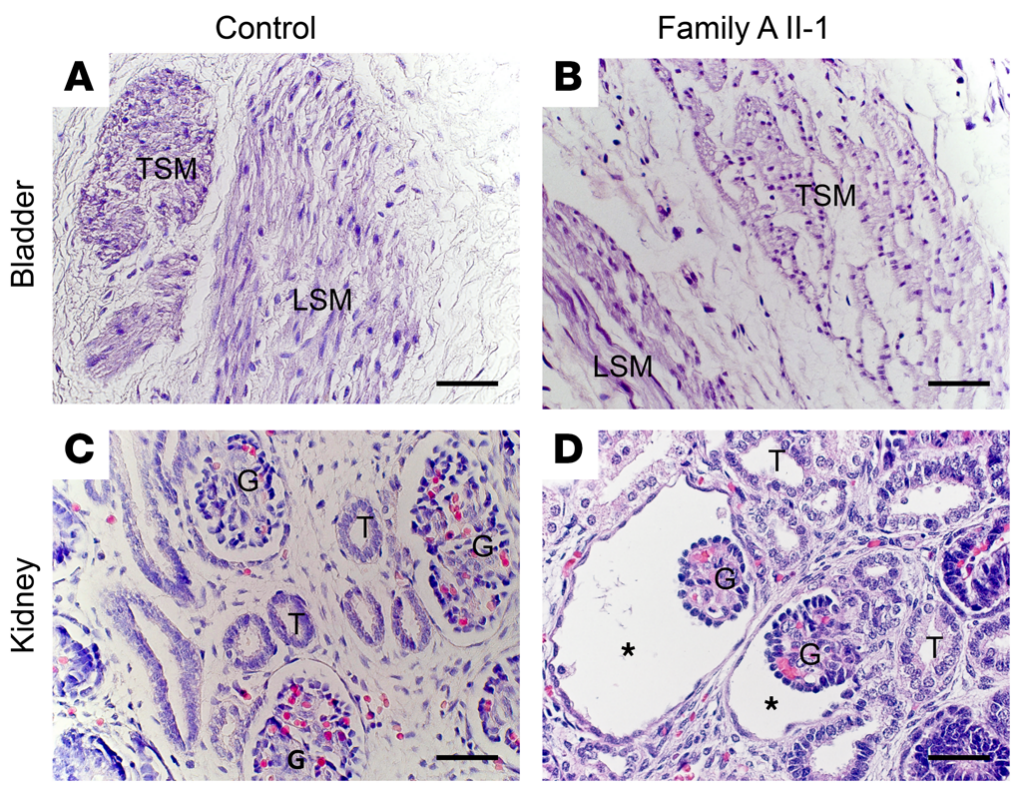
Bladder and kidney abnormalities in family A. (**A** and **C**) From healthy midgestation fetuses. (**B** and **D**) From affected fetus from family A. H&E staining from urinary bladders shows transverse sections of muscle bundles (TSM) and longitudinal sections of muscle bundles (LSM) in the healthy and affected fetuses. Note, however, that the bundles in the affected fetus appear disorganized and less compact compared with the well-defined muscle fibers in the control. (**C**) In a control fetal kidney, glomeruli (G) and tubules (T) are evident. (**D**) In the kidney from the affected fetus, glomeruli are cystic, with dilated Bowman’s spaces (asterisks), a characteristic of fetal urinary flow obstruction. Scale bars: 20 μm.

The index patient of family A (II-2) (Figure 1A) had a history of antenatal megabladder, and a bladder diverticulum was surgically resected in infancy. At 32 years of age, cardiac evaluation showed noncompaction cardiomyopathy and marked dilation (51 mm) of the aortic root. She also had an atrial septal defect, a ventricular septal defect (VSD), a patent ductus arteriosus, and a bicuspid aortic valve. Her brother (II-1) had been diagnosed prenatally with megabladder and VSD, prompting clinical termination of pregnancy at midgestation. Autopsy revealed PBS, and histology showed disorganized SM bundles in the bladder and glomerular cysts in the kidneys (Figure 2). The distal urethra was patent (Supplemental Figure 1; supplemental material available online with this article; https://doi.org/10.1172/JCI128545DS1), but the prostate and proximal urethra were not identified. A conclusion regarding anatomical obstruction was not possible because formal tests of urethral patency were not undertaken. The hindgut contained only a defined circular SM layer (Supplemental Figure 2) and lacked the longitudinal layer that should be present at this gestational age. In contrast, 2 normal SM layers were present in the small intestine and pulmonary artery SM appeared normal (Supplemental Figure 2). Ultrasonography revealed no bladder or heart abnormalities in the parents. Whole-exome sequencing (WES) in index patient II-2 and Sanger sequencing in II-1 determined that both siblings carried compound heterozygous variants in *MYOCD* (p.[S229Qfs*17];[E530G], respectively called family A mutation 1 and family A mutation 2 in Figure 1A). The variant p.[S229Qfs*17] is predicted to create a premature stop codon and was paternally inherited, while the missense variant p.[E530G] is located in the functional leucine zipper (LZ) of the encoded protein (12, 13) and was maternally inherited (Figure 1, A and B). Both variants were absent from over 120,000 control exomes (14). WES in the index case failed to reveal pathogenic variants in genes known to cause megabladder, including *ACTG2*, *CHRM3*, *HPSE2*, *LRIG2*, smooth muscle myosin heavy chain 11 (*MYH11*), and myosin light chain kinase (*MYLK*) (3, 15–17).

Next, we ascertained 22 additional families with megabladder or PBS of unknown etiology, identifying 7 affected individuals from 3 unrelated families, all with heterozygous predicted loss-of-function *MYOCD* variants (Figure 1A and Supplemental Table 1). In family B, there were 3 male fetal deaths, all with PBS (Figure 1, A and C). WES revealed a heterozygous variant, c.343C>T, in these 3 brothers, predicted to result in a premature stop p.[R115*]. This variant was also present in the unaffected mother, unaffected maternal grandmother, and a healthy female sibling, each with normal bladder and heart imaging. The grandmother (II-3) reported a male stillbirth (III-4) of unknown cause in the third trimester. She had 5 siblings, including 4 brothers who died antenatally (II-5, II-6, II-7, and II-8), each with a megabladder (further details unavailable), and 1 healthy sister (Figure 1A). Family C’s first pregnancy was terminated after diagnosis of PBS in the male fetus (Figure 1, A and C). Chromosomal microarray analysis revealed a heterozygous de novo 420 kb deletion (chr17p12;hg19:12,172,568-12,609,597) encompassing the first 2 exons of *MYOCD*, including the start codon. In family D, 3 males had PBS: 2 died prenatally (III-1 and IV-1), and the other (III-4) was born and underwent kidney transplantation for end-stage renal failure (Figure 1A). Sanger sequencing of *MYOCD* identified a heterozygous deletion of a single base c.1053-1054del, resulting in a predicted frameshift p.[N351Kfs*19].

**Figure 3:**
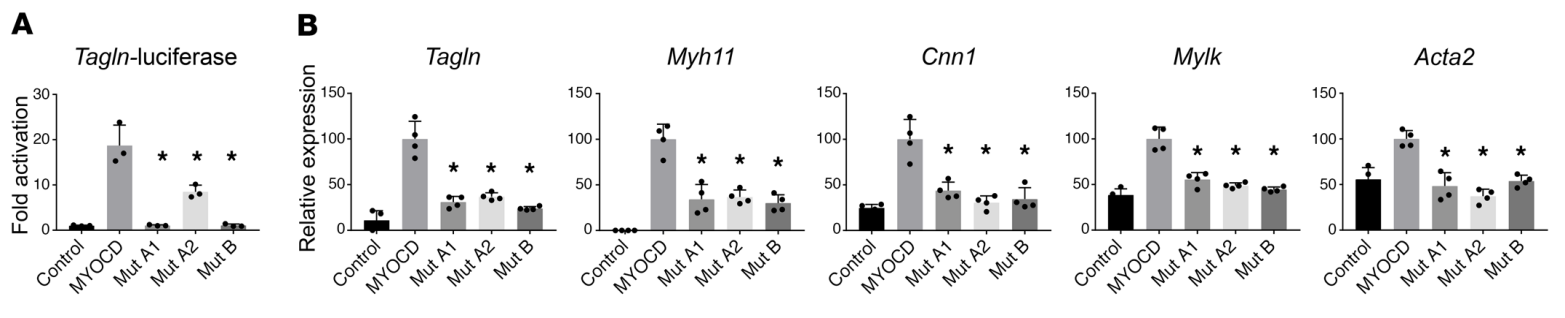
*MYOCD* mutations abrogate activation of SM cell gene expression in vitro. (**A**) Mouse fibroblasts were transiently transfected for 48 hours with expression vectors for *MYOCD* or the indicated *MYOCD* mutants (mutation A1: p.S229Qfs*17; mutation A2: p.E530G; mutation B: p.R115*) and a luciferase reporter linked to the Transgelin (*Sm22*) promoter (*n* = 3/group). (**B**) Mouse fibroblasts were transfected with expression plasmids encoding *MYOCD* or the indicated mutants (*n* = 4/group). An empty expression plasmid served as a control. RNA was isolated, and SM gene expression was measured by qPCR. *GAPDH* was used to normalize expression. Overexpression levels of *MYOCD* were comparable between conditions (Supplemental Figure 3). *P < 0.01 compared with WT *MYOCD* according to 1-way ANOVA with Dunnett’s multiple comparison test. Shown are representative experiments of 2 independent repeats.

We tested the abilities of proteins encoded by *MYOCD* variants from families A and B to activate the promoter of transgelin (*Sm22* or transgelin [*Tagln*]), encoding a SM contractile protein. Western blotting revealed that the 2 predicted nonsense variants (p.[S229Qfs*17] mutation A1 from family A and p.[R115*] mutation B from family B) produced a truncated protein, whereas the missense variant (E530G, mutation A2 from family A) produced full-length protein (Supplemental Figure 3 and Supplemental Table 3). Neither nonsense variant resulted in activation of the *Tagln*-luciferase reporter, whereas the missense variant resulted in diminished activity versus WT *MYOCD* (Figure 3A). The above results regarding the missense variant are consistent with previous reports showing that *MYOCD* homodimerizes through the LZ domain and that homodimerization facilitates stronger activation of SRF-dependent reporter genes (8, 12). *Myocd* is normally not expressed in 10T1/2 fibroblasts, but experimental overexpression in these cells activates the SM differentiation program (8). We transfected 10T1/2 cells with either WT *MYOCD* or one of each of the 3 variants from families A and B. As assessed by quantitative PCR (qPCR), WT *MYOCD* strongly induced endogenous expression of the SM transcripts *Tagln*, *Myh11*, Calponin 1 (*Cnn1*), *Mylk*, and SM actin alpha 2 (*Acta2*). Conversely, each of the 3 *MYOCD* variants resulted in either a lack of increased expression or statistically significantly blunted responses (Figure 3B).

**Figure 4:**
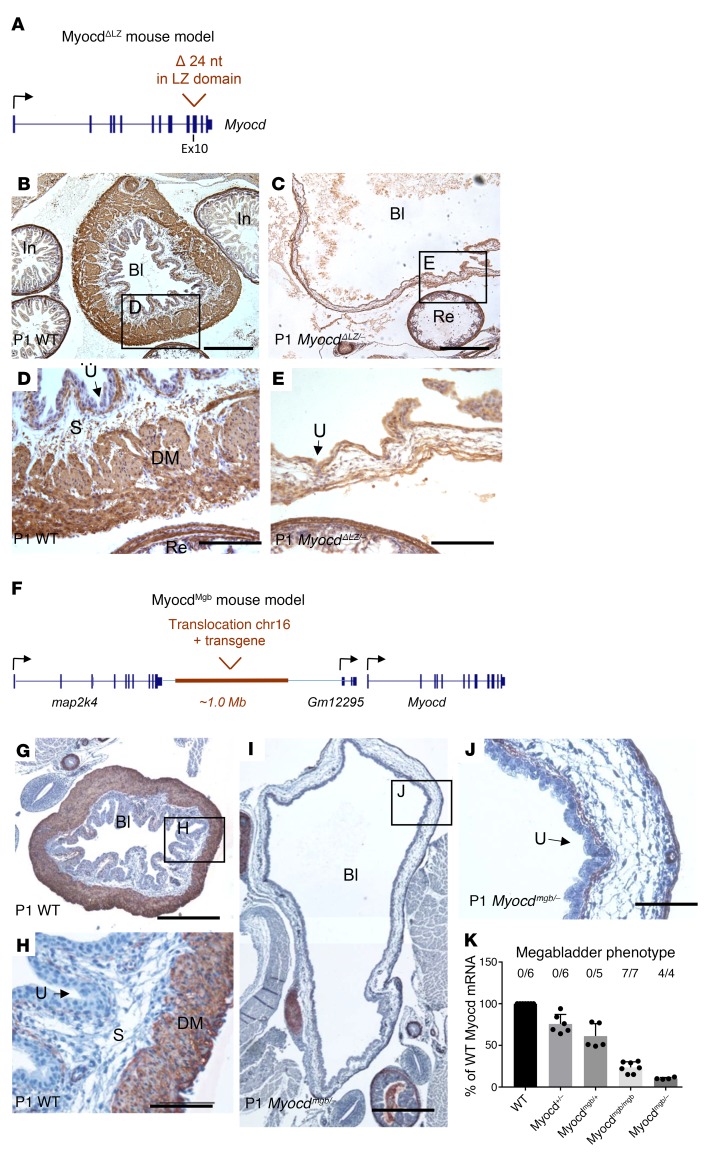
*Myocd* loss of function in mice causes the megabladder phenotype. (**A**) Schematic representation of the *Myocd^ΔLZ^* allele, in which 24 nucleotides are deleted in the LZ domain (p.I531_R539delinsM in NP_666498.2). (**B**–**E**) αSMA immunohistochemistry in 1-day-old neonates from *Myocd^+/–^* and *Myocd^ΔLZ/+^* crosses. (**C**) Compound heterozygosity (*Myocd^ΔLZ/–^*; reminiscent of the alleles present in the affected individuals in family A) results in wall thinning of the bladder and lack of SM cells compared with the WT bladder wall (**B**). (**D** and **E**) Higher magnifications of WT and *Myocd^ΔLZ/–^* bladder walls showing lack of αSMA-expressing muscle bundles in the putative detrusor layer, although expression appeared retained in myofibroblast-like cells in the lamina propria directly below the urothelium and in the rectum. (**F**) Schematic representation of the *Myocd^Mgb^* allele. (**G**–**J**) Representative αSMA immunohistochemistry in P1 bladder of WT (**G** and **H**) and *Myocd^mgb/–^* compound heterozygote (**I** and **J**) (from a cross of *Myocd^mgb/+^* and *Myocd^+/–^* mice). Note the severe bladder distention and absent detrusor muscle in the *Myocd^mgb/–^* bladder. Bl, bladder; In, intestine; Re, rectum; U, urethelium; S, submucosa; DM, detrusor muscle. Scale bars: 500 μm (**B**, **C**, **G**, and **I**); 100 μm (**D**, **E**, **H**, and **J**). (**K**) *Myocd* mRNA levels were quantified by qPCR using E15 bladders of WT, *Myocd^+/–^*, *Myocd^mgb/+^*, *Myocd^mgb/mgb^*, and *Myocd^mgb/–^* mice. The absolute numbers of embryos developing megabladder as a fraction of the total number of embryos analyzed are indicated above the graph and reveals a highly penetrant phenotype in the *Myocd^mgb/mgb^* and *Myocd^mgb/–^* mice.

To assess whether reduced MYOCD activity causes megabladder, we took advantage of a newly generated mutant mouse line carrying an allele, *Myocd^ΔLZ^*, in which critical residues within the LZ of Myocd were specifically deleted (Figure 4A and Supplemental Figure 4A). Using primers that distinguish the WT from the LZ mutated transcript, we showed that both were detected in neonatal bladders of *Myocd^ΔLZ/+^* mice (Supplemental Figure 4B). We crossed *Myocd^ΔLZ/+^* mice with those carrying a null allele of *Myocd* (*Myocd^+/–^*) (18). The alleles in the compound mutant offspring (*Myocd^ΔLZ/–^*) therefore mimicked the LZ and nonsense mutant alleles in family A. In contrast to homozygous null *Myocd* mutants (*Myocd^–/–^*) (9), compound heterozygous mutant offspring (*Myocd^ΔLZ/–^*) survived to birth. In line with the human urinary tract malformations, *Myocd^ΔLZ/–^* mice developed grossly dilated bladders with little or no SM in their walls (Figure 4, A–E, and Supplemental Figure 4, C and D). In *Myocd^ΔLZ/–^* mouse bladders, transcript levels of several *Myocd* target genes (i.e., *Acta2*, *Myh11*, *Mylk*, *Tagln*, and *Cnn1*) were blunted versus those of WTs (Supplemental Figure 4E).

To gain further insight into the potential role of *Myocd* gene dosage in bladder malformations, we examined the megabladder mouse (*mgb*) generated by random insertion and translocation of a transgene into chromosome 11 (19). Transcriptional profiling in the bladders of these mice had already revealed that levels of *Myocd* transcripts were significantly reduced (20). Here, we identified the translocation breakpoint (together with 4 copies of translocated chr. 16 region) approximately 500 kb upstream of the *Myocd* gene (Figure 4F, Supplemental Figure 5, and Supplemental Table 4), suggesting the presence of a regulatory *Myocd* enhancer. Next, we crossed *Myocd^mgb/+^* with *Myocd^+/–^* mice (9) and demonstrated that compound mutant offspring (*Myocd^mgb/–^*) had megabladders, providing further evidence that marked reduction of *Myocd* causes this phenotype (Figure 4, G–J, and Supplemental Figures 6 and 7). In addition, we observed patent ductus arteriosus in newborn *Myocd^mgb/–^* mice, but not in *Myocd^+/–^* mice with just 1 allele mutated (Supplemental Figure 8). By studying various *Myocd* mutants (Figure 4K), we showed that a 70% to 80% reduction in *Myocd* mRNA in the bladder is sufficient to produce megabladder in mice. Interestingly, neonatal *Myocd^mgb/–^* mouse bladders have severely reduced transcript levels of *Myocd* target genes, yet in aortas and hearts of these same mice, the levels of Myocd target transcripts (apart from *Myh11* in the aorta) are similar to those of heterozyous (*Myocd^mgb/+^*) controls (Supplemental Figure 9).

The bladder phenotypes in families carrying *MYOCD* variants are thus mimicked by 2 distinct mouse models with reduced *Myocd* activity. Supplemental Table 2 gives an overview of the phenotypes observed in the mouse lines. The collective results demonstrate that MYOCD plays a unique role in proper development of the bladder wall. Reduced *Myocd* activity resulted in little or no SM differentiation in the mouse bladders and in disorganized SM bundles in the human fetal bladder. These SM defects would diminish the muscular force required to void urine from the bladder, resulting in the functional equivalent of lower urinary tract obstruction with severe bladder distension, ultimately culminating in kidney failure and death (19, 21).

Megabladder/PBS is a sex-limited trait with 95% male predominance, likely the result of differences in urethra and bladder development and length differences in urethra between males and females (4, 6). Additionally, sex hormones may play a role in defining the severity and progression of the disease, as clinical evidence demonstrates increased male susceptibility to acute and chronic kidney injury (22, 23). Indeed, in our study, 7 of 8 males proven to carry heterozygous loss-of-function *MYOCD* variants died before birth, whereas all 6 female carriers with heterozygous loss-of-function variants appeared healthy. Consistent with this observation, male *Myocd^mgb/mgb^* mice were also more severely affected than females (Supplemental Table 2 and ref. 19). Notably, the only female with bladder disease in our study carried biallelic variants in *MYOCD*, suggesting that further reduction in *MYOCD* levels is needed to cause bladder phenotypes in females. This is supported by both *Myocd* mouse models in which compound heterozygosity (i.e., *Myocd^mgb/–^* or *Myocd^LZ/–^*) caused megabladder in either sex (Supplemental Table 2). An intriguing feature is the incomplete penetrance of bladder disease in a healthy male *MYOCD* mutation carrier in family D. This may be caused by allelic imbalance, where the penetrance of a dominant loss-of-function mutation is determined by the expression level of the second allele, for instance, due to variants in promoter or enhancer regions of *MYOCD*. Alternatively, redundancy with other MYOCD family members, such as MRTF-A, which is also expressed in developing bladders (24), may affect penetrance.

Overall, we propose that we have identified a semidominant disorder (25) in which heterozygous loss-of-function variants in *MYOCD* cause congenital megabladder, while biallelic loss-of-function *MYOCD* variants also cause a cardiovascular phenotype. Both biallelic carriers developed congenital heart defects, while the affected female was found, when investigated as an adult, to have severe dilation of the aortic root. A similar association of cardiac defects was observed in the *Myocd^mgb/–^* mice (ref. 26 and Supplemental Figure 8). Notably, a previous study already hinted at the possible involvement of MYOCD in megabladder, as it described one sporadic case with PBS and a 1.3 Mb deletion of multiple genes, including *MYOCD* (27). Other SM-related genes have been implicated in PBS. These include variants in *ACTA2*, a MYOCD target gene, as well as *MYH11* and *MYLK*, in which variants can cause visceral myopathy, a phenotype encompassing megabladder (3, 16). Moreover, each of these 3 genes has been associated with inherited thoracic aortic aneurysm and dissection (28). Notably, an SM-restricted deletion of *Myocd* in mice causes dilation of several visceral organs, including the bladder, as well as dilation of the aorta (11). Hence, there is compelling evidence that reduced MYOCD levels can result in urological and cardiovascular disease.

In conclusion, we demonstrate for what we believe is the first time that variants in *MYOCD* result in human disease. We propose that monoallelic loss-of-function variants in *MYOCD* cause congenital megabladder in males and that biallelic variants are associated with disease manifest in females that also involves the cardiovascular system. These findings not only have important implications for genetic counseling of families with megabladder, but also shed light on bladder development and expand the pathophysiological spectrum of inherited SM disorders.

## Methods

Experimental procedures are provided in Supplemental Methods.

### Study approval.

Blood samples for genetic testing were obtained upon written consent. Informed consent for DNA studies, clinical records, and use of ultrasound pictures and histological analysis of the terminated fetus of family A was obtained. Control human embryonic material, collected with maternal consent and ethical approval (REC 08/H0906/21+5 and REC 18/NE/0290), was sourced from the MRC and Wellcome Trust Human Developmental Biology Resource. Mice were maintained according to the NIH *Guide for the Care and Use of Laboratory Animals* (National Academies Press, 2011). All experiments using *Myocd^ΔLZ^* mice complied with federal and institutional guidelines and were reviewed and approved by the UCSF IACUC. The Mgb mouse studies were approved by the IACUC of the Nationwide Children’s Hospital**.**

## Author contributions

ACH, GMB, and AVP share first authorship, and the order in which they are listed has been determined by workload. ACH, GMB, AVP, WGN, ASW, and EEC designed the study. TBG, FML, IVDM, AMP, MLR, KDW, RG, LC, and ED performed experiments. ACH, EO, SR, KDL, and FB contributed clinical samples and clinical data. ACH, JME, GMB, AVP, and WGN performed genetic analysis. ACH, GMB, AVP, KMM, ARJ, BLB, WGN, ASW, and EEC analyzed experimental data. BLB, KMM, and MLR contributed mouse lines. EJMH and VMC were instrumental in interpretation of the data. ACH, AVP, ASW, and EEC wrote the manuscript.

## Supplementary Material

Supplemental data

## Version 1 09/12/2019

In-Press Preview

## Version 2 11/04/2019

Electronic publication

## Version 3 12/02/2019

Print issue publication
