# Supplementary material for: Loss-of-function variants in myocardin cause congenital megabladder in humans and mice
Source: J Clin Invest. 2019 Nov 4;129(12):5374–80. doi: 10.1172/JCI128545 (PMC6877301; doi:10.1172/JCI128545)
Supplement: Supplemental data [file jci-129-128545-s216.pdf]

## Online methods

### Patients

Patients were identified at the department of Clinical Genetics of Amsterdam UMC, location VUmc, The Netherlands (Family A), the department of Medical Genetics, Ospedale San Salvadore, L'Aquila, Italy (Family B), and the department of Clinical Genetics, UMC Utrecht, the Netherlands (Family C and D). Peripheral blood samples from patients and their unaffected first-degree relatives for genetic testing were obtained upon written consent. Informed consent for DNA studies, the use of clinical records and ultrasound pictures and histological analysis of the terminated foetus of family A was obtained. Control human embryonic material, collected with maternal consent and ethical approval (REC 08/H0906/21+5), was sourced from the MRC and Wellcome Trust Human Developmental Biology Resource (http://www.hdbr.org/).

### Histology of human tissue

Tissues were fixed in 4% paraformaldehyde and sectioned at 5 µm. After dewaxing and rehydration, endogenous peroxidase was quenched by incubation with hydrogen peroxide. Haematoxylin and eosin staining was performed according to standard procedures.

### Whole-exome sequencing family A, C, D

WES and variant calling were conducted using BWA-MEM (0.7.5), GenomeAnalysisTK-2.8–1-g932cd3a, Cartagenia Bench Lab NGS (Agilent Technologies) and the SeqCap EZ MedExome (NimbleGen; Roche, Madison, Wisconsin, USA) on a HiSeq2000 (Illumina, San Diego, California, USA). Variants were filtered and analysed as described previously(1). Candidate variants were confirmed by Sanger sequencing. WES was performed in the three probands; Sanger sequencing was performed in all their available relatives. Informed consent was obtained in all cases. Identified variants have been submitted to Clinvar.

**Whole-exome sequencing family B**

Target DNA enrichment was designed to capture all exons and 40 base pair (bp) of the intron/exon boundaries and was achieved using the Agilent SureSelectXT custom Kit. NGS was performed on the Illumina HiSeq® 2500 platform according to manufacturer's protocol. Sequence data was mapped to the hg19 reference human genome using the Burrows-Wheeler aligner software (version 0.6.2; http://bio-bwa.sourceforge.net). Genome Analysis Tool Kit software (version 2.4.7; https://www.broadinstitute.org/gatk) was used for recalibration of base quality score and for insertion-deletion realignment before using the unified genotyper (https://www.broadinstitute.org/gatk) for variant calling. The average sequencing coverage was 95.13% at 20x and 90.41% at 30x. Analysis of exome data was performed using VarSeq™ v2.2 (Golden Helix, Inc., Bozeman, MT, www.goldenhelix.com). Identified variant has been submitted to Clinvar.

**Array CGH family C**

Array-CGH analysis was performed on proband and parents using Agilent 180K oligo-array (Amadid 023363, Agilent, Santa Clara, CA), with 13-kb overall median probe spacing and a GRCh37/hg19 browser. Standard methods were used for labeling and hybridization. Samples were hybridized against a pool of 40 healthy sex-matched human reference samples. Data were analyzed with Genomic Workbench 6.5 (Agilent) and Cartagenia [BENCHlab CNV v5.0 (r6643); Agilent]. Identified variant has been submitted to Clinvar.

**Expression vectors, cell culture, tranfection**

A human WT MYOCD expression construct was kindly provided by Dr. J. Miano (University of Rochester, NY, USA). The WT MYOCD open reading frame was subcloned into pCRII-TOPO vector (Invitrogen, Carlsbad, CA, USA) to introduce the following mutations, p.S229Qfs*17 (Family A1), p.E530G (Family A2) and p.Arg115* (Family C), using the QuickChange Site-directed mutagenesis kit (Agilent Technologies, Santa Clara, CA, USA) according to the manufacturer's instructions. All products were confirmed by sequencing of the complete

MYOCD open reading frame. After successful introduction of the mutations, WT MYOCD and MYOCD mutants were subcloned into pcDNA3.1 for overexpression experiments. Mutagenesis primers are shown in supplemental Table 3.

To activate endogenous smooth muscle gene expression by MYOCD and the mutants, MYOCD constructs were transfected in mouse fibroblasts (CH3-10T1/2 cells, ATCC), which were cultured in DMEM supplemented with 10% FBS, 1% penicillin–streptomycin and 200 mM L-glutamine (all from Gibco, Life Technologies) in a humidified incubator at 37 °C with 5% $CO_2$. 1 µg pcDNA-MYOCD plasmid was transfected per well of a 6-well plate using Genejammer (Stratagene) according to manufacturer's instruction at 50-75% confluence. 48 hours after transfection, cells were harvested for RNA isolation using TRIzol (Invitrogen, Carlsbad, CA, USA) according to the manufacturer's instructions.

**Quantitative PCR**

For gene expression analysis in the 10T1/2 cells and P1 mouse bladders, heart and aorta, ~1 µg RNA was treated with DNAse I (Invitrogen, Carlsbad, CA, USA) and cDNA was synthesized using Superscript II RT (Invitrogen, Carlsbad, CA, USA). Quantitative PCR (qPCR) was performed on a Lightcycler 480 (Roche Diagnostics, Risch-Rotkreuz, Switzerland) using SYBR green (Roche Diagnostics, Risch-Rotkreuz, Switzerland). Gene expression was normalized to Gapdh expression. Analysis of qPCR data was performed using LinRegPCR analysis software(2).

For gene expression analysis in E15 mouse bladders, cDNA was synthesized from ~300ng RNA using the QuantiTect Reverse Transcription kit (Qiagen, Cat. No. 205313). Quantitative PCR (qPCR) was performed on a QuantStudio 5 (ThermoFisher Scientific) using PowerUp Sybr Green Master Mix (ThermoFisher Scientific, Cat. No. A25741). Gene expression was normalized to Gapdh expression. Analysis of qPCR data was performed using LinRegPCR analysis software(2). Primers used are included in Supplemental Table 3.

**Semi-quantitative RT-PCR**

Neonatal mouse bladders were frozen in liquid nitrogen and the frozen tissue was crushed with a pestle. The resulting powder was resuspended in Buffer RLT from the RNeasy Micro Kit (Qiagen, Cat. No. 74004), passed through a QiaShredder column (Qiagen, Cat. No. 79654), and RNA was isolated according to the RNeasy Micro Kit protocol. One microgram total RNA was used as input for reverse transcription using the QuantiTect Reverse Transcription Kit (Qiagen, Cat. No. 205313) according to the manufacturer's protocol. To control for genomic DNA contamination, one reaction was set up using 1µg RNA from MyocdΔLZ/+ bladder with all reaction components except the reverse transcriptase enzyme (-RT). One microliter from each RT reaction was used as template for PCR using the primers LZ-fwd (5'-acggacgagagtctgctgag-3'), LZ-rev (5'-ctgcagctgctcttctgcttc-3'), and GAPDH fwd (5'-ggtggacctcatggcctaca-3'), GAPDH rev (5'-ctctcttgctcagtgtccttgct-3') as an internal control in each reaction. Samples were taken from each reaction after the indicated number of cycles and run on a 2% agarose/TBE gel for 80 minutes at 85V.

**SRF-dependent luciferase reporter assays**

The Sm22-luciferase reporter plasmid harbors a 1434 bp promoter region of Sm22 (Tagln) containing two SRF binding sites(3). 24-well plates with CH3-10T1/2 cells were transfected at ~50% confluency with 100 ng pcDNA-Myocd, 75 ng Sm22-luciferase reporter, 5 ng pCGN-SRF and 30 ng CMV-renilla per well, using the transfectant Genejammer (Stratagene). After 48 hours, luciferase assays were performed using the luciferase assay system (Promega) according to the manufacturer's instructions. All MYOCD constructs were expressed at a comparable level as confirmed by qRT-PCR (Supplemental Figure 3). Measurements were performed in triplicate and repeated two times.

**Generation of *Myocd*$^{\Delta LZ}$ mice**

The *Myocd*$^{\Delta LZ}$ allele was generated by CRISPR-Cas9 genome editing at the Cardiovascular Research Institute, University of California, San Francisco (UCSF), CA as previously described(4). Briefly, candidate spCas9 guide sequences were identified within a 200bp region of the mouse genome encompassing the coding sequence of the *Myocd* leucine zipper (LZ) domain. The guide sequence 5'-aagtgatcaaccagctcacc(tgg)-3' (PAM sequence indicated in parentheses) was selected based on predicted high on-target and low off-target effects. Single-guide RNA (sgRNA) *in vitro* transcription template was generated by annealing a forward oligonucleotide containing a T7 RNA polymerase promoter (underlined) and guide sequence (bold) (5'-taatacgactcactatagggg**aagtgatcaaccagctcacc**gttttagagctagaa-3') with a reverse oligonucleotide containing the remainder of the sgRNA sequence (5'-aaaaaaagcaccgactcggtgccactttttcaagttgataacggactagccttattttaacttgctatttctagctctaaaac-3'). Annealed oligonucleotides were filled in using Klenow fragment (NEB, M0210) and PCR amplified. sgRNA was transcribed using the MEGAshortscript T7 kit (Life Technologies, AM1354) and then purified using the MEGAclear kit (Life Technologies, AM1908). Purified sgRNA and humanized *Streptococcus pyogenes* Cas9 (hspCas9) mRNA (SBI, CAS500A-1) were co-injected into the cytoplasm of fertilized mouse oocytes using standard techniques as described previously(5). F0 founders were screened for editing events by PCR amplification and BclI digestion of an 839bp fragment encompassing the targeted genomic region using the following primers: *Myocd* LZ genotyping F, 5'-tgctgctgggtgaagagac-3' and *myocd* LZ genotyping R, 5'-agtgtcaggcaccaagacag-3'. BclI digestion of this fragment from a wild type allele results in two fragments of ~400bp, whereas the edited allele was predicted to be resistant to BclI digestion owing to the coincidence of the BclI site with the guide sequence. One F0 male founder carried a 24bp deletion, leading to loss of 9 amino acids (INQLTWKLR) and the creation of one amino acid (Met) from the encoded protein (p.I531_R539delinsM in NP_666498.2), including one isoleucine and two leucine residues previously shown to be critical for myocardin dimerization(6). This founder was outcrossed to wild type females to establish the F1 generation. A single F1 male carrying the 24bp deletion was used to establish the *Myocd*$^{\Delta LZ}$ line, and a colony was generated by outcrossing to wild type animals on a mixed

genetic background. Animals were maintained according to the NIH Guide for the Care and Use of Laboratory Animals.

### Histology of *Myocd*$^{\Delta LZ}$ mice and *Myocd* knockout mice

*Myocd*$^{\Delta LZ/+}$ males were crossed to female mice harboring a *Myocd* gene knock out allele (*Myocd*$^{+/-}$)(7). Neonates were collected shortly after birth and euthanized by decapitation with tail tissue collected for PCR genotyping. Whole neonates were fixed in 4% formaldehyde, embedded in paraffin, and 7µm transverse sections were obtained using a microtome and affixed to glass slides. Serial sections were stained with haemotoxylin and eosin or processed for immunohistochemistry as follows: Following deparaffinization and rehydration, heat-mediated antigen retrieval was performed in citrate buffer. Staining was performed using the Vector ImmPRESS Excel Staining Kit (Vector Labs, MP-7601) according to the manufacturer's instructions using alpha smooth muscle actin antibody (Abcam, ab5694) or Rabbit IgG isotype control (Cell Signaling Technology, #3900) diluted 1:100 in PBS + 0.1% Tween-20 with 2.5% Horse serum. Following 3,3'-Diaminobenzidine (DAB) staining, sections were counterstained with haemotoxylin and mounted using VectaMount Medium (Vector Laboratories). The megabladder phenotype was defined by megacystis at autopsy, and on histology, finding both a lack of SM-like cells and immunostaining for αSMA.

### Genomic organization of the megabladder (mgb) mouse model

Generation of the Mgb mouse model has been described(9). In short, the MLR19 transgene was randomly inserted into chromosome 16 between 26.6 and 27.5 Mb, and in conjunction with a portion of chromosome 16, the transgene then translocated into a second domain on chromosome 11, resulting in the development of megabladder in homozygous mice(9). Triple fluorescence in situ hybridization (FISH) analysis was used to narrow the translocation breakpoint on chromosome 11 (Suppl. Figure 5). FISH using 8 BACs from chromosome 11 (Supplemental Table 4) and an Mgb transgene positive BAC clone was performed to screen and map the Mgb interchromosomal insertional translocation site under guidance of the

Molecular Cytogenetics core at The Ohio State University as described(9). Briefly, bone marrow was aspirated from the femur of transgenic and wild-type mice and grown overnight in RPMI medium supplemented with 15% FBS. Cells were harvested using standard cytogenetics methods and stored in Carnoy's fixative at -20°C. BAC DNA for fluorescence *in situ* hybridization analysis was isolated using the Spin Doctor BAC Prep kit according to the conditions outlined by the supplier (Gerard Biotech, Oxford, OH). BAC probes were labeled with Spectrum Orange, Spectrum Green, or Spectrum Aqua using a nick translation kit according to the manufacturer's recommendations (Vysis, Downer Groves, IL). BAC DNA was combined with mouse Cot-1 DNA, ethanol precipitated, resuspended in hybridization buffer (Sigma), and denatured. Probes were applied to fresh denatured slides, and hybridization was carried out overnight at 37°C. Slides were washed in 2xSSC/0.1% NP-40 for 5 min at 42°C. Fluorescence signals were viewed with a Zeiss Axioscope 40 microscope and analyzed with the Applied Imaging System (Zeiss, Thornwood, NY). For G-banding pattern, the fixed cell suspension was dropped onto precleaned, warm, wet slides. The slides were aged at 90°C for 1 h, banded with trypsin, and stained with Wright's stain. Banded metaphase chromosomes were analyzed using a Zeiss Axioskop 40 and an Applied Imaging Karyotyping System.

Comparative Genomic Hybridization (CGH) services were performed by Nimblegen. Genomic DNA from wildtype and homozygous mice was prepared from liver samples using Gerard Biotech kit according to manufacturer's recommendations. Samples were labeled (Cy3-reference, and Cy5-test) and hybridized according to standard procedures. Samples were hybridized to mouse CGH 385K whole-genome Custom tiling array (26_mm8_wg_cgh, NimbleGen System, Inc.). The custom mouse array design included a median probe spacing of 5000bp with an average of 45-85bp probe length. Genomic tracks were visualized using SignalMap software.

**Complementation studies of the Mgb mouse model and *Myocd* knockout mice**

Since we mapped the translocation breakpoint of the transgene in the Mgb mouse model approximately 500 kb upstream of *Myocd*, we named the Mgb allele: *Myocd*$^{mgb}$. Complementation studies were performed using mice harboring a *Myocd* gene knock out allele (*Myocd*$^{+/-}$)(8) and the *Myocd*$^{mgb/mgb}$ to yield Mendelian ratio litters where ½ of the litter was compound heterozygous (*Myocd*$^{mgb/-}$). Expected litter sizes were maintained and embryos and postnatal pups were collected for evaluation. Animals were maintained according to the NIH Guide for the Care and Use of Laboratory Animals. Approval number and institution for these mouse studies: IACUC#02105AR, Nationwide Children's Hospital, Columbus, Ohio, USA.

**Myocd In Situ Hybridization.**

In situ hybridization for Myocd expression was performed as previously described(10). Briefly, E15 embryos from timed matings of *Myocd*$^{mgb/+}$ mice were collected and specimens were fixed and processed using standard procedures. Serial paraffin-embedded transverse sections (10 uM) of the bladder were affixed to slides. In situ hybridization using $^{35}$S-UTP radiolabeled riboprobes was performed by prehybridizing sections with hybridization solution for 90 minutes while riboprobes were labeled with $^{35}$S-UTP. The riboprobes were purified with a NucAway kit (Amersham) and the slides were hybridized with 70,000 DPM counts of riboprobe in hybridization solution. Slides were incubated O/N at 50$^{o}$C in a hybridization oven. Following incubation, the slides were washed in high stringency washes (FSM, STE x2, STE + yeast-tRNA and RNase, STE + BME, FSM x2, 2x SSC, 0.1x SSC) and dehydrated. Slides were air-dried, placed on film, and exposed for 3 days. Following exposure, slides were emulsion coated (Kodak), incubated at 4$^{o}$C for 10–14 days, and developed. Lastly, slides were cover slipped with permaslip and observed under dark field and fluorescence. The myocardin in situ probe used was derived from published exon sequences as previously described and annotated(10).

**Statistical analysis**

Data are presented as the mean value ± standard deviation. Measurements were taken from distinct samples. Statistical tests used to compare between conditions are indicated in the figure legends.

**Data availability**

All data generated or analysed during this study are included in this published article (and its supplementary information files).

**References**


1. Heinen CA et al. Mutations in *TBL1X* Are Associated With Central Hypothyroidism. *J Clin Endocrinol Metab* 2016;101(12):4564–4573.

2. Ruijter JM et al. Amplification efficiency: linking baseline and bias in the analysis of quantitative PCR data. *Nucleic Acids Res* 2009;37(6):e45–e45.

3. Wang D et al. Activation of cardiac gene expression by myocardin, a transcriptional cofactor for serum response factor.. *Cell* 2001;105(7):851–62.

4. Wang H et al. One-step generation of mice carrying mutations in multiple genes by CRISPR/Cas-mediated genome engineering.. *Cell* 2013;153(4):910–8.

5. Anderson JP et al. HRC is a direct transcriptional target of MEF2 during cardiac, skeletal, and arterial smooth muscle development in vivo.. *Mol Cell Biol* 2004;24(9):3757–68.

6. Wang Z, Wang D-Z, Pipes GCT, Olson EN. Myocardin is a master regulator of smooth muscle gene expression.. *Proc Natl Acad Sci U S A* 2003;100(12):7129–34.

7. Huang J et al. Myocardin regulates expression of contractile genes in smooth muscle cells and is required for closure of the ductus arteriosus in mice.. *J Clin Invest* 2008;118(2):515–25.

8. Li S, Wang D-Z, Wang Z, Richardson JA, Olson EN. The serum response factor coactivator myocardin is required for vascular smooth muscle development. *Proc Natl Acad Sci* 2003;100(16):9366–9370.

9. Singh S et al. Identification of a Unique Transgenic Mouse Line That Develops Megabladder, Obstructive Uropathy, and Renal Dysfunction. *J Am Soc Nephrol* 2007;18(2):461–471.

10. DeSouza KR, Saha M, Carpenter AR, Scott M, McHugh KM. Analysis of the Sonic Hedgehog Signaling Pathway in Normal and Abnormal Bladder Development. *PLoS One* 2013;8(1):e53675.

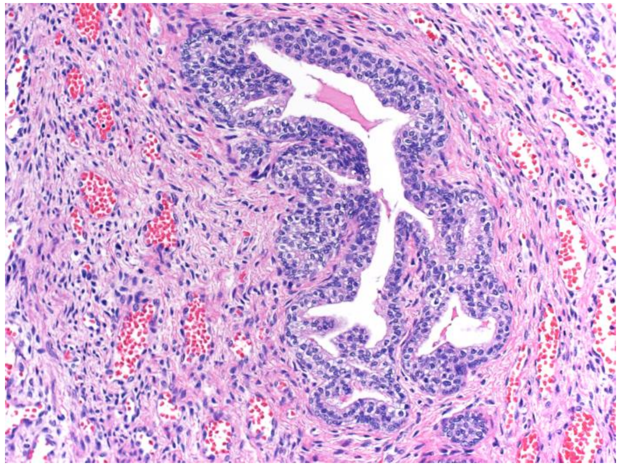

**Supplemental Figure 1. Patency of the urethra in the affected male fetus (II-1) of family A.** H&E staining of urethra in the affected fetus of family A showing the absence of an anatomical blockage.

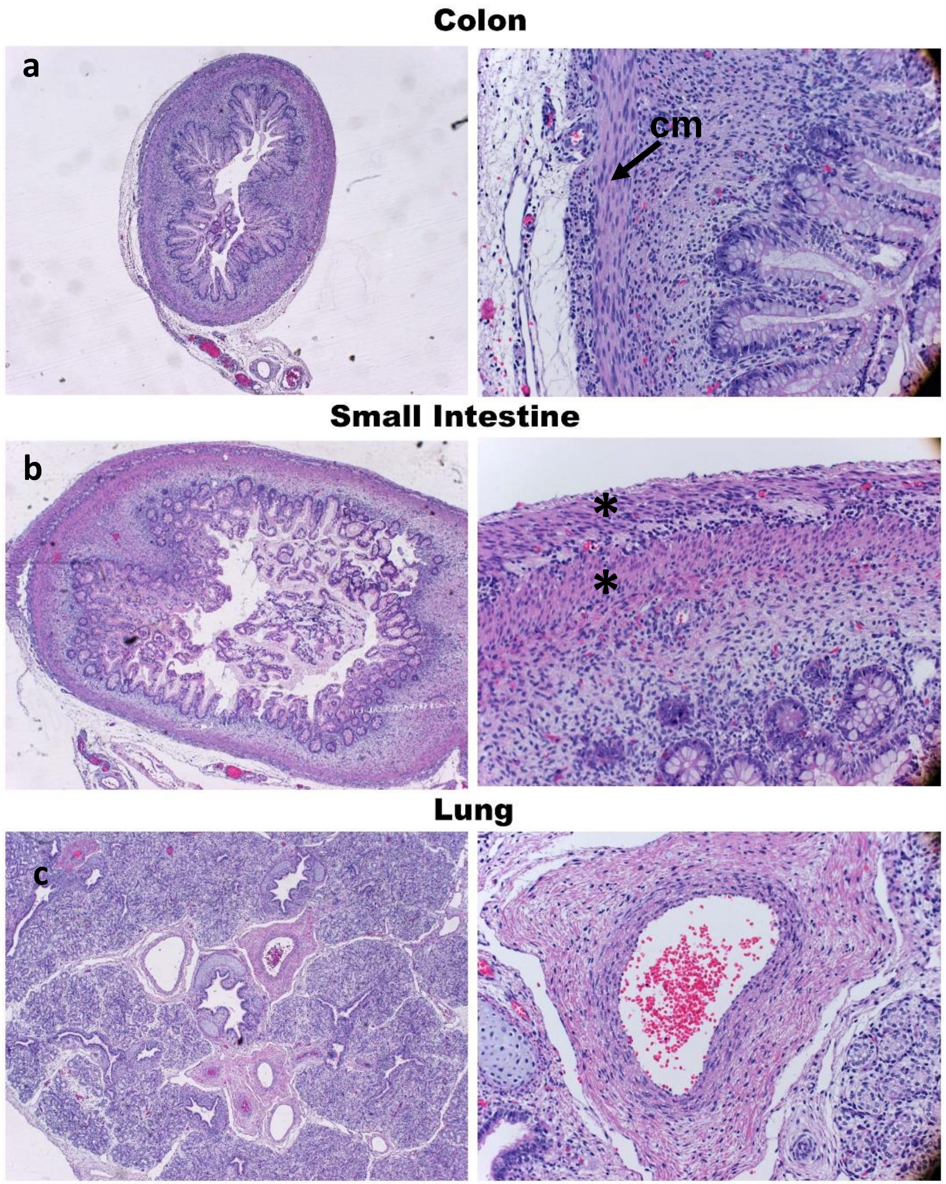


**Supplemental Figure 2. Hematoxylin and Eosin stained histology section of extra-renal viscera in the affected male fetus (II-1) of family A.** Boxes on left are low power views, while boxes on right are high power views. **(a)** The colon displays only a defined circular muscle layer (cm) and lacks the longitudinal layer that should be present at this gestational age **(b)** The small intestine, in contrast, has the expected two muscle layers indicated by asterisks. **(c)** A large pulmonary vessel with robust muscle layer in its wall.

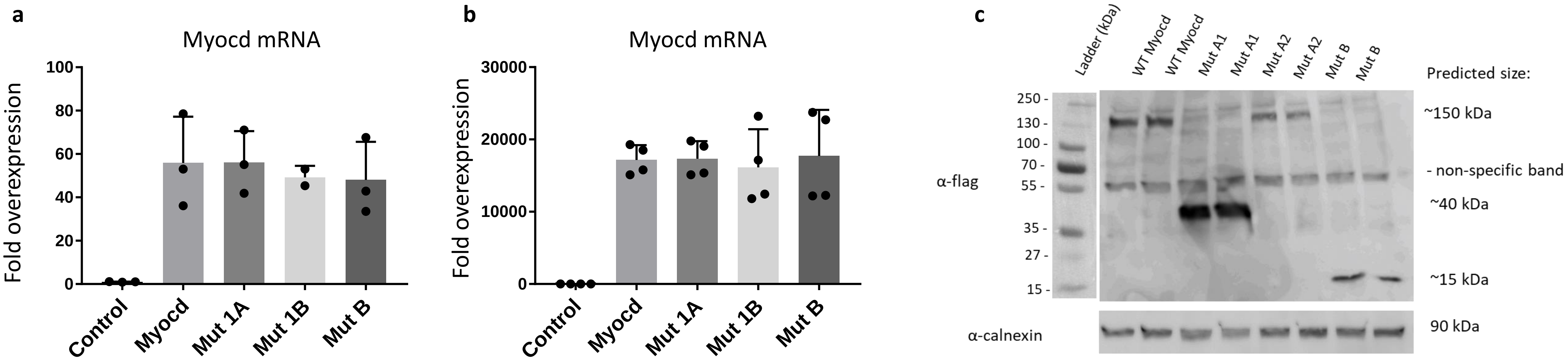


**Supplemental Figure 3. *MYOCD* mRNA and protein after overexpression of indicated *MYOCD* mutants in 10T1/2 and Hek293T cells**. **a**, *Myocd* mRNA levels measured by qRT-PCR in the Sm22-luciferase experiment as depicted in Figure 3a. **b**, *Myocd* mRNA overexpression levels in experiment shown in figure 3b. *Myocd* expression was normalized for GAPDH expression. **c,** Western blotting in lysates of HEK293T cells after overexpression of wild-type Myocd and indicated Mycod mutants. All Myocd constructs contain a flag-tag at their N-terminus and were detected using an anti-flag antibody (anti-FLAG M2 antibody, 1:500, #200472 Agilent Technologies). As shown, both nonsense mutants expressed truncated proteins at the expected size (40 kDa for Mut A1 and 15 kDa Mut B), while the missense variant (Mut A2) expressed full length Myocd (~150kDa). Of note, 48 hours after transfection, cells were treated for 6 hours with 10 nM of MG132 to prevent degradation of proteins with premature stops. Interestingly, without MG132 treatment, we could not detect the 15 kDa band of mut B, whereas both other mutant proteins were readily detected. This indicates that the truncated protein derived from Mut B is degraded by the proteasome. Calnexin was detected (Calnexin antibody AF18, 1:500, Santa Cruz sc-23954) as a loading control.

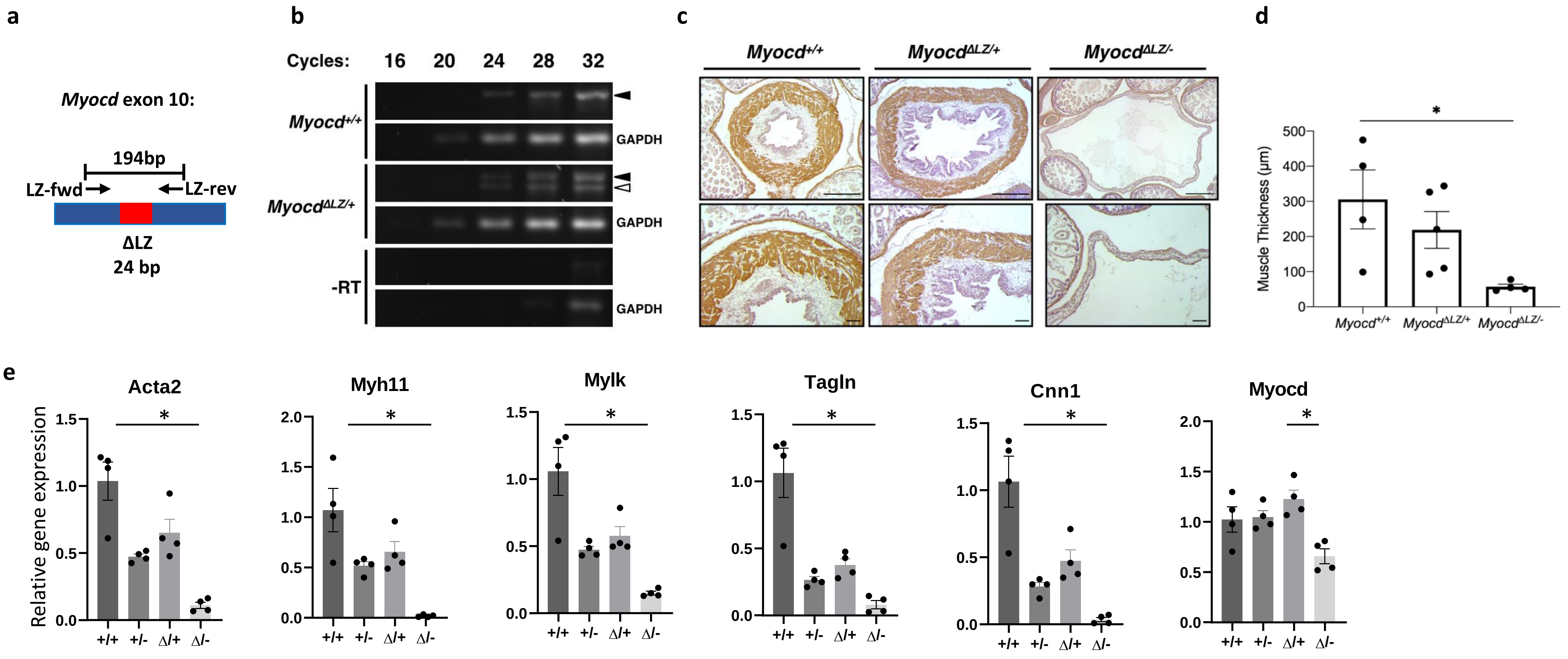


**Supplemental Figure 4. Gene expression analysis and bladder wall thickenss measurements in Myocd$^{ΔLZ}$ mice. (a)** Schematic of *Myocd* exon 10, with the 24bp ΔLZ mutation highlight in red. PCR primers were designed on either side of the deletion site, resulting in an expected product size of 194 bp from the WT allele and 170 bp from the ΔLZ allele. **(b)** RNA was extracted from WT and *Myocd*$^{ΔLZ/+}$ bladder at P0. cDNA was synthesized and used as a template in PCR using LZ-fwd and LZ-rev primers, as well as Gapdh primers as an internal control. In *Myocd*$^{ΔLZ/+}$ bladders, WT (filled arrowhead) and ΔLZ (open arrowheads) transcripts appear to be present at comparable levels indicating that *Myocd*$^{ΔLZ}$ transcripts are stably expressed in neonate bladders. (c) αSMA IHC was performed on bladders of postnatal day 1 WT, Myocd$^{ΔLZ/+}$ and Myocd$^{ΔLZ/-}$ mice. **(d)** Thickness of the bladder wall was measured on 13 sites per bladder on average. Number of animals per group: WT (n=4), Myocd$^{ΔLZ/+}$ (n=5) and Myocd$^{ΔLZ/+}$ (n=4). * p=0.034 One Way ANOVA with Tukey's multipe comparison test. **(e)** qPCR for Myocd and smooth muscle gene expression in the bladder of indicated genotypes at embryonic day 15. Graphs show mean +/- SEM, and expression is corrected for Gapdh. Number of animals per group: WT (+/+, n=4), Myocd$^{+/-}$ (+/-, n=4), Myocd$^{ΔLZ/+}$ (Δ/+, n=4) and Myocd$^{ΔLZ/+}$ (Δ/-, n=4). * p<0.05 according to Kruskal-Wallis test with Dunn's multiple comparison correction. Interestingly, Myocd$^{ΔLZ/+}$ embryonic bladders tended to have modest downregulations of these same transcripts, but their bladder walls contain SM on histology (panel e) and the thickness of these walls was not significantly less than wild-types (panel d).

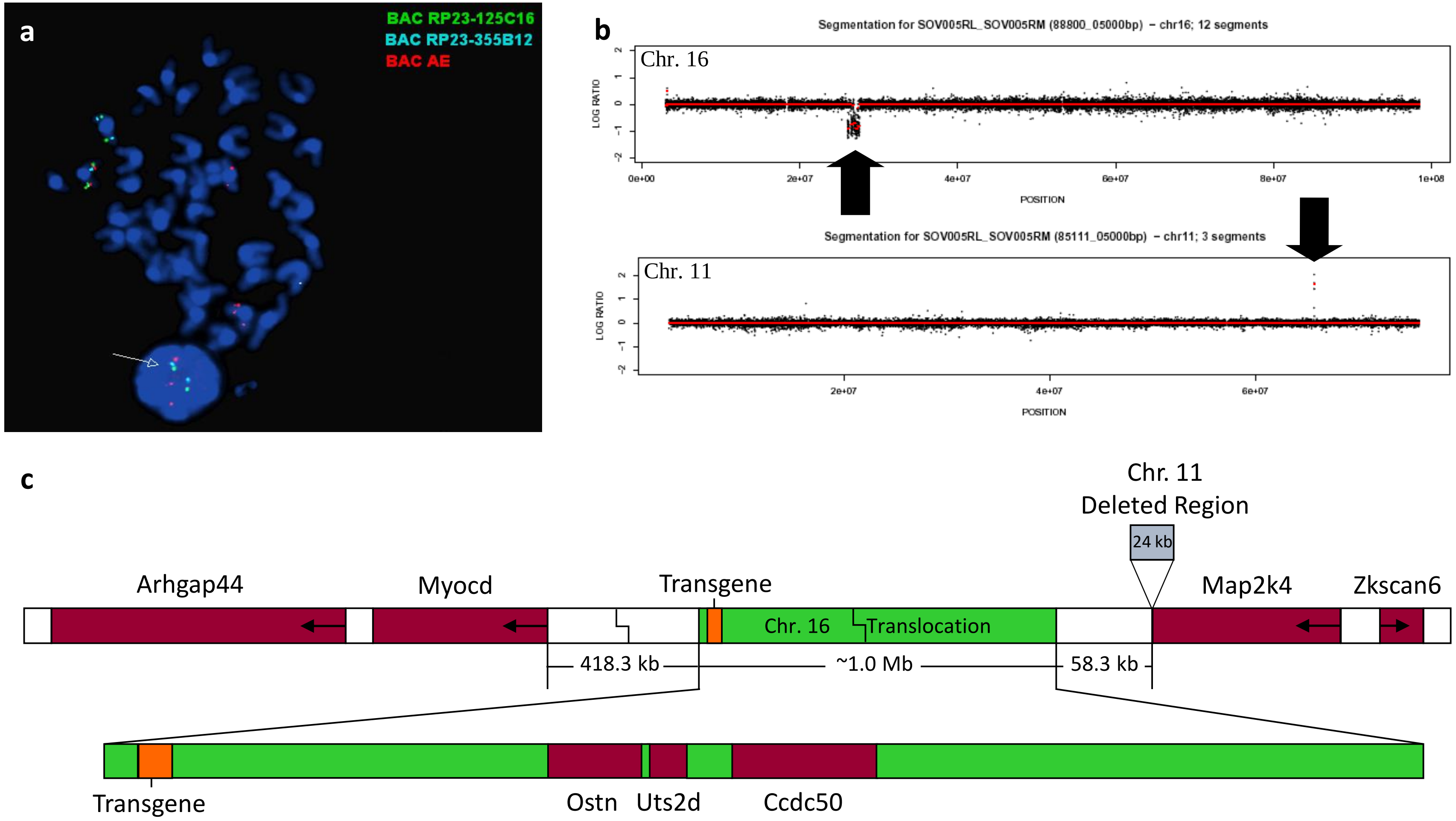


**Supplemental Figure 5. *Myocd* genomic locus in the megabladder mouse model** (**a**) Triple fluorescence *in situ* hybridization (FISH) analysis narrowed the translocation breakpoint on Chromosome 11 to 65215576 – 66268441. (**b**) NimbleGen comparative genomic hybridization (CGH) identified four copies of the translocated Chromosome 16 region and a 24 kb deleted region on Chromosome 11. (**c**) Genomic organization of the mgb locus on Chromosome 11 showing associated genes including Rho GTPase Activating Protein 44 (**Arhgap44**), Myocardin (**Myocd**), Mitogen-Activated Protein Kinase Kinase 4 (**Map2k4**), Zinc Finger with KRAB and SCAN Domains 6 (**Zkscan6**), Osteocrin (**Ostn**), Urotensin 2 Domain Containing (**Uts2d**) and Coiled-Coil Domain Containing 50 (**Ccdc50**).

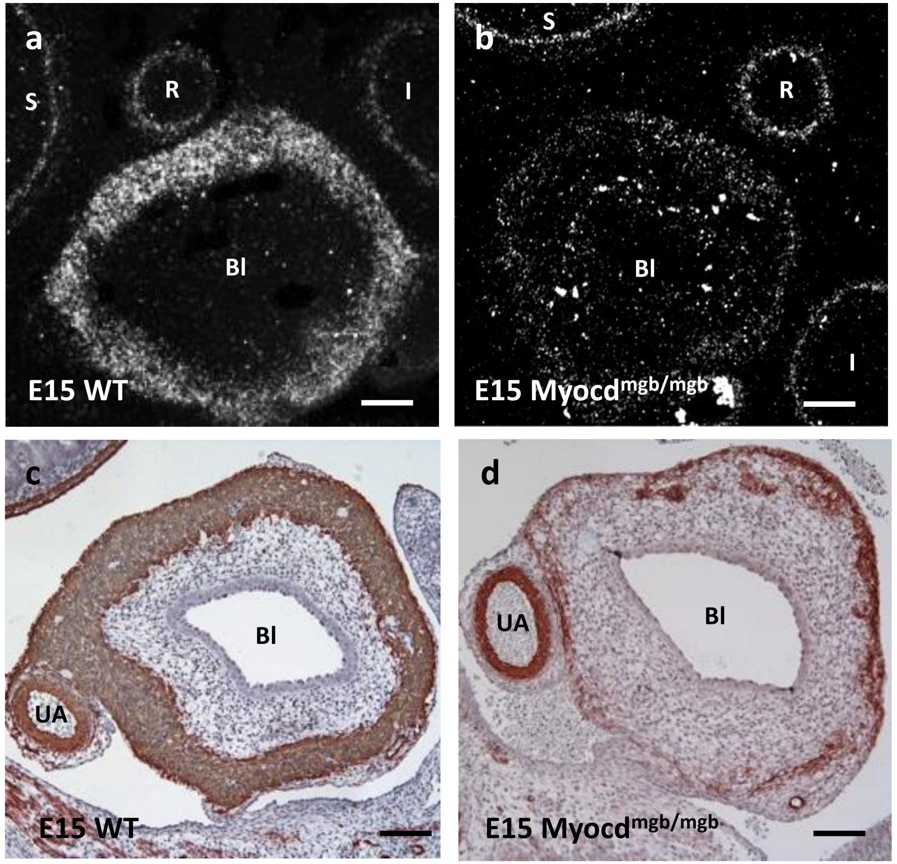


**Supplemental Figure 6.** *In situ* hybridization (**a,b**) and immunohistochemistry (**c,d**) of embryonic day 15 WT (**a,c**) and *Myocd$^{mgb/mgb}$* (**b,d**) mice showing Myocd mRNA (**a,b**) and αSMA protein (**c,d**) expression in the bladder (**Bl**), stomach (**S**), intestine (**I**), rectum (**R**) and umbilical artery (**UA**). At this specific developmental stage in mice, detrusor smooth muscle is being formed. Note specific loss of Myocd mRNA *and* αSMA protein expression in *Myocd$^{mgb/mgb}$* bladders versus WT, while Myocd expression within the stomach, intestine and rectum appears similar. Scale bars are 100 µm.

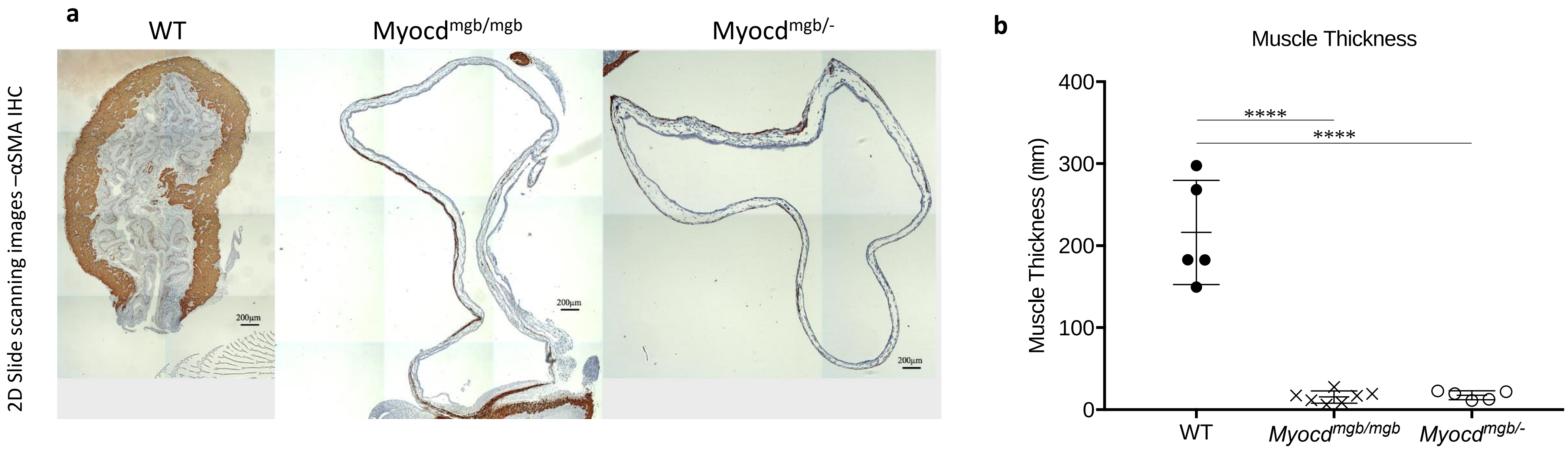


**Supplemental Figure 7. Bladder wall thickness quantifications in Myocd$^{Mgb/-}$ mice, at postnatal day 1. (a)** αSMA IHC was performed on bladders of WT, Myocd$^{mgb/mgb}$ and Myocd$^{mgb/-}$ mice. **(b)** Thickness of αSMA-positive detrusor smooth muscle was measured on 13 sites per bladder on average. Number of animals per group: WT (n=5), Myocd$^{mgb/mgb}$ (n=5) and Myocd$^{mgb/-}$ (n=7). **** $p<0.0001$ One Way ANOVA Tukey multipe comparison correction.

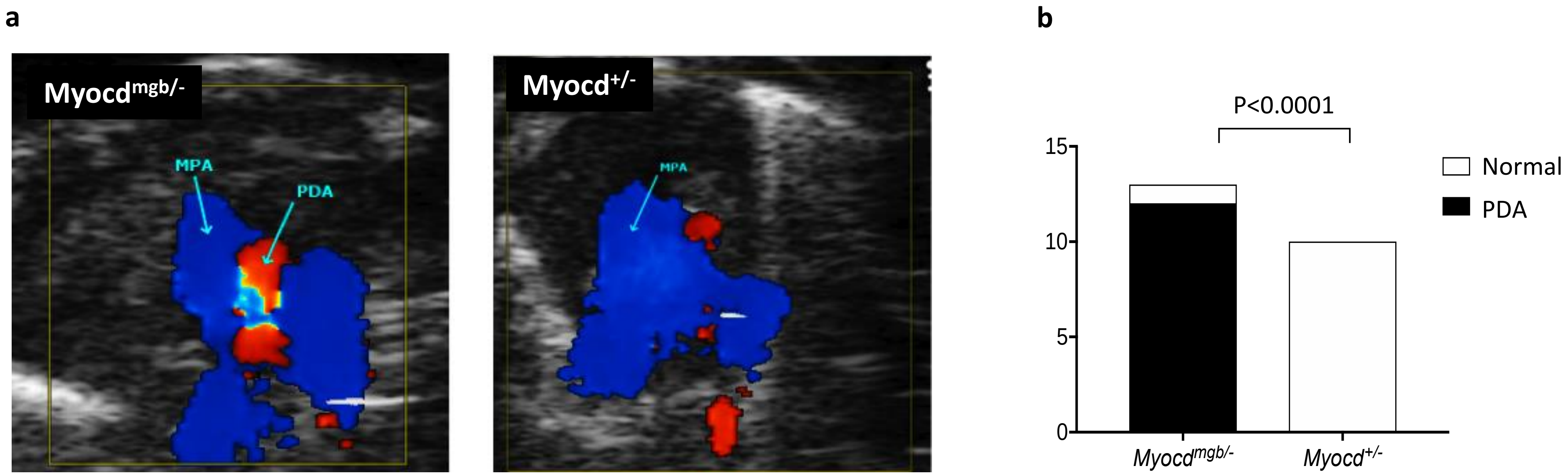


**Supplemental Figure 8. (a)** 2D transthoracic echocardiogram from postnatal day 1 showing patent ductus arteriosus (PDA) in $Myocd^{mgb/-}$ mice versus $Myocd^{+/-}$ mice. **(b)** 12 out of 13 $Myocd^{mgb/-}$ neonates, and 0 out of 10 $Myocd^{+/-}$ mice developed PDA. P-value by Fisher Exact test.

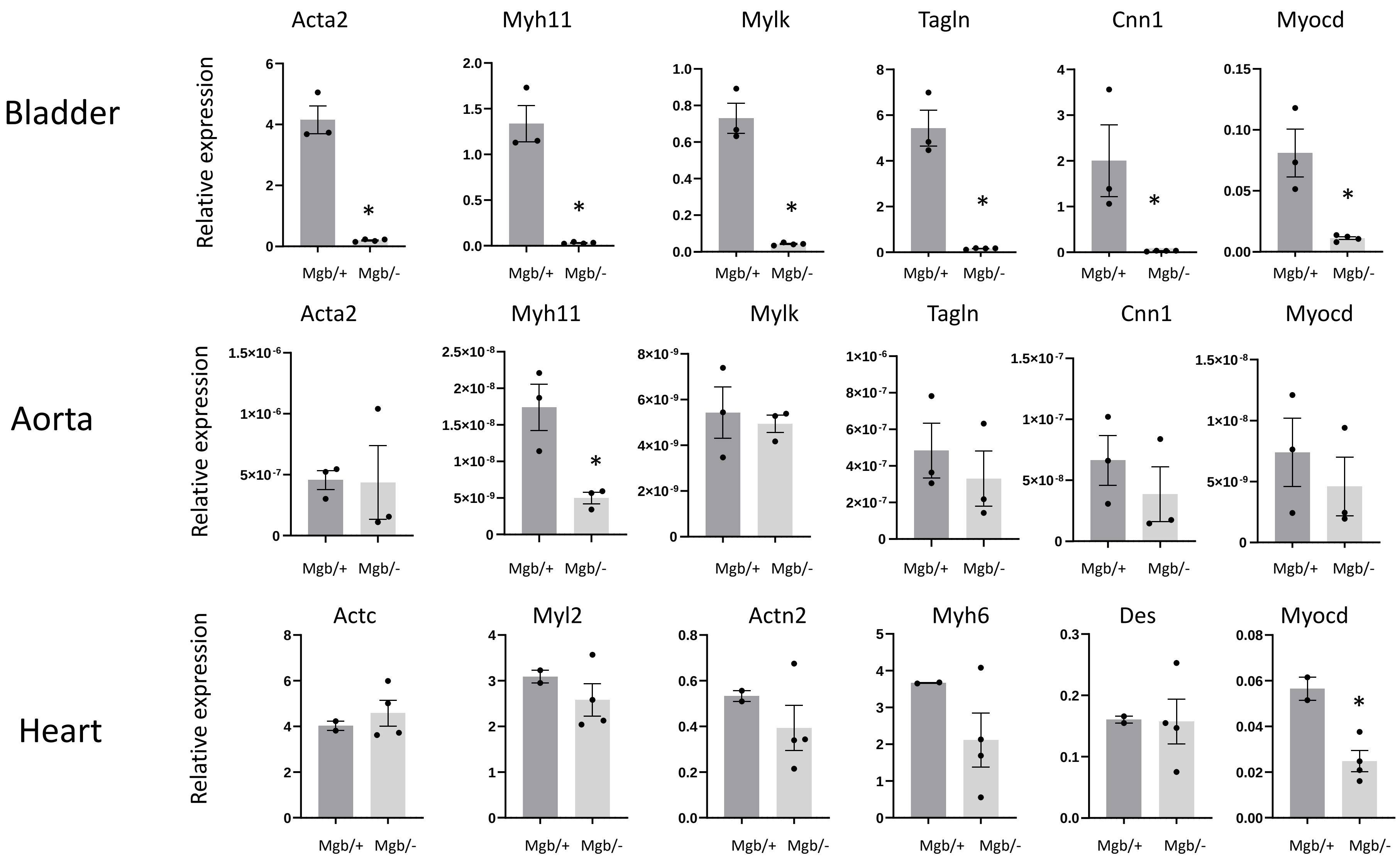


**Supplemental Figure 9. Expression of Myocd-target genes is blunted in bladder, but not in heart and aorta of $Myocd^{Mgb/+}$ and $Myocd^{Mgb/-}$ neonates.** Smooth muscle target genes (Acta2, Myh11, Mylk, Tagln and Cnn1) were measured in the bladder and the aorta, and the cardiac target genes of Myocd (Actc, Myl2, Actn2, Myh6 and Des) were measured in hearts using qRT-PCR. Note that Myocd targets and Myocd expression are mainly downregulated in the bladder. Expression is corrected for Gapdh. Number of animals per group: $Myocd^{mgb/+}$ (n=2-3) and $Myocd^{mgb/-}$ (n=3-4). * $p<0.05$ by Student's t-test.

| | | | | | | Cardiac abnormalities | | | | | | Urogenital abnormalities | |
|---|---|---|---|---|---|---|---|---|---|---|---|---|---|
| | **Member** | **Gender** | **Status** | **Genotype** | **Affected** | **ASD** | **BAV** | **NCCM** | **PDA** | **TAA** | **VSD** | **Bladder** | **Kidney** |
| *Family A* | I-1 | M | Alive | p.[S229Qfs*17] | - | - | - | - | - | - | - | - | - |
| | I-2 | F | Alive | p. [E530G] | - | - | - | - | - | - | - | - | - |
| | II-1 | M | Deceased | p.[S229Qfs*17]; [E530G] | + | - | - | - | - | - | + | + | Glomerular cysts at autopsy |
| | **II-2** | **F** | **Alive** | **p.[S229Qfs*17]; [E530G]** | **+** | **+** | **+** | **+** | **+** | **+** | **+** | **MB, diverticulum** | **-** |
| *Family B* | II-1 | F | Alive | p.[R115*] | - | | | | | | | - | - |
| | II-3 | F | Alive | p.[R115*] | - | - | - | - | - | - | - | - | - |
| | II-5 | M | Deceased | Unknown | +˜ | | | | | | | MB˜ | |
| | II-6 | M | Deceased | Unknown | +˜ | | | | | | | MB˜ | |
| | II-7 | M | Deceased | Unknown | +˜ | | | | | | | MB˜ | |
| | II-8 | M | Deceased | Unknown | +˜ | | | | | | | MB˜ | |
| | III-1 | M | Alive | WT | - | | | | | | | - | - |
| | III-3 | F | Alive | p.[R115*] | - | - | - | - | - | - | - | - | - |
| | III-4 | M | Deceased | Unknown | +˜ | | | | | | | MB˜ | |
| | IV-1 | M | Deceased | p.[R115*] | + | - | - | - | - | - | - | MB | Increased parenchymal echogenicity and initial calico-pyelic microcystic dilatation |
| | IV-2 | M | Deceased | p.[R115*] | + | | | | | | | MB | |
| | **IV-3** | **M** | **Deceased** | **p.[R115*]** | **+** | **-** | **-** | **-** | **-** | **-** | **-** | **MB** | **Increased parenchymal echogenicity** |
| | IV-4 | F | Alive | p.[R115*] | - | - | - | - | - | - | - | - | - |
| Family C | I-1 | M | Alive | WT | - | | | | | | | | |
| | I-2 | F | Alive | WT | - | | | | | | | | |
| | **II-1** | **M** | **Deceased** | **420kb deletion** | **+** | | | | | | | **MB** | |
| Family D | II-2 | F | Alive | p.[N351Kfs*19] | - | | | | | | | | |
| | II-3 | M | Alive | p.[N351Kfs*19] | - | | | | | | | - | - |
| | III-1 | M | Deceased | p.[N351Kfs*19] | + | | | | | | | MB | |
| | III-3 | F | Alive | p.[N351Kfs*19] | - | | | | | | | | |
| | **III-4** | **M** | **Alive** | **p.[N351Kfs*19]** | **+** | | | | | | | **MB** | **Congenital agenesis right kidney, end-stage renal failure, underwent renal transplant** |
| | III-5 | F | Alive | Unknown | - | | | | | | | - | - |
| | IV-1 | M | Deceased | p.[N351Kfs*19] | + | | | | | | | MB | |
| | IV-2 | M | Alive | Unknown | - | | | | | | | - | |

**Supplemental Table 1. Clinical and genetic features of the four families with congenital megabladder**

ASD, atrial septal defect; BAV, bicuspid aortic valve; F, female; M; male, MB; megabladder, diagnosed with ultrasound and/or autopsy; NCCM, non-compaction cardiomyopathy; PDA, patent ductus arteriosus; TAA, thoracic aortic aneurysm; VSD, ventricular septal defect; WT, wild type

+ clinical feature is present; - clinical feature is absent. If it is uncertain if a clinical feature was present nothing is filled in.

~ Stillbirth with external features consistent with prune belly syndrome

The probands are depicted in bold

**Supplemental Table 2. Phenotypes of the studied mouse models.**

| Genotype | Megabladder | Cardiac Defects | Longevity |
|---|---|---|---|
| **Wildtype** | No | No | Normal |
| **Myocd$^{mgb/+}$** | No | No | Normal |
| **Myocd$^{+/-}$** | No | No | Normal |
| **Myocd$^{-/-}$** | Die before detectable | Yes | Embryonic Lethal (E9.5) |
| **Myocd$^{mgb/mgb}$** | Yes (♂and ♀) | No | ♂ : die within 6 wks postnatally<br>♀: Normal* |
| **Myocd$^{mgb/-}$** | Yes (♂ and ♀) | Yes (PDA) | Die within 1 week (♂ and ♀) |
| **Myocd$^{LZ/-}$** | Yes (♂ and ♀) | No obvious cardiac abnormalities by gross anatomical inspection | Die within 1 week (♂ and ♀) |

* Females of the Myocd$^{mgb/mgb}$ genotype do develop as prominent megacystis as male Myocd$^{mgb/mgb}$, but they don't have the shortened life span that males exhibit. The longer life span in females may relate to shorter urethra in females, which may enable drops of urine to be expelled over time or sexually dimorphic hormones may play a role in modulating kidney disease progression. Either way, the female kidneys are not as severely affected by the functional obstruction, therefore they don't die of renal failure at the same time that males do.

PDA: patent ductus arteriosus.

**Supplemental Table 3: Primers for mutagenesis and qPCR**

| Application | Primer name | Primers sequence<br>Forward (5'->3') | Primers sequence<br>Reverse (5'->3') |
|---|---|---|---|
| Mutagenesis | p.S229Qfs*17 | TATGGGGGTGCTGGGGGGGGCC AAG | CTTGGCCCCCCCCAGCACCCCC ATA |
| Mutagenesis | p.E530G | GGAGTTTCCAGGTGAGTCCATTG ATCACCTTCTGC | GCAGAAGGTGATCAATGGACTCA CCTGGAAACTCC |
| Mutagenesis | p.R115* | AGTGGCCCTGGTCATAGAGCAAT TTTTTCATTGAGATC | GATCTCAATGAAAAAATTGCTCTA TGACCAGGGCCACT |
| qPCR | Human MYOCD | ACTGCAGAGAGGTCCATTCC | TTCACTTCGAGTCTGATCCG |
| qPCR | Human GAPDH | ACCCACTCCTCCACCTTTGAC | ACCCTGTTGCTGTAGCCAAATT |
| qPCR | Mouse Tagln (sm22) | ACGATGGAAACTACCGTGG | TGCGAGGCTCCTCTGTTGC |
| qPCR | Mouse Myh11 | AGACACTGGAAGCCATGTC | AGCTGAAGGACAGATGATAC |
| qPCR | Mouse Cnn1 | ACAAGTTCAGTCCACTCTCC | AGTCCAATGATGTTCCTGCC |
| qPCR | Mouse Acta2 | TGTGGCTATTCAGGCTGTGC | AGGCACGTTGTGAGTCACAC |
| qPCR | Mouse Mylk | TCCGTGATCTGGAAGTCGTG | TTGAACCAGACCACCTCAGGG |
| qPCR | Mouse Gapdh | GGTGGACCTCATGGCCTACA | CTCTCTTGCTCAGTGTCCTTGCT |
| qPCR | Mouse Desmin | GTGGATGCAGCCACTCTAGC | TTAGCCGCGATGGTCTCATAC |
| qPCR | Mouse Myh6 | TGAGTGGGAGTTTATCGACTTCG | CCTTGACATTGCGAGGCTTC |
| qPCR | Mouse Actn2 | CATCGAGGAGGATTTCAGGAAC | CAATCTTGTGGAACCGCATTTT |
| qPCR | Mouse Actc1 | CTGGATTCTGGCGATGGTGTA | CGGACAATTTCACGTTCAGCA |
| qPCR | Mouse Myl2 | CACCGTGTTCCTCACGATGTT | CCTTGAATGCGTTGAGAATGGTC |
| qPCR | Mouse Myocd | CCAGCCCCCATCCTATGAAGA | CATCGGCTGGCATTTCTCCAC |
| RT-PCR | Mouse Myocd LZ | ACGGACGAGAGTCTGCTGAG | CTGCAGCTGCTCTTCTGCTTC |

Note: Mutated bases are underlined.

**Supplemental Table 4. BACs Utilized for Translocation Mapping in the Megabladder mouse model.**

| Serial No. | Name of BAC | Chromosomal Band Location | Gene Location on Chromosome | Role |
|---|---|---|---|---|
| 1. | RP23-1016 | B1.1 | 43062893-43073047 | Screening of transgene on chromosome 11 |
| 2. | RP24-336H1 | B2 | 95339826 - 95516805 | Screening of transgene on chromosome 11 |
| 3. | RP23-180B18 | B2 | 59694511 - 59695427 | Screening of transgene on chromosome 11 |
| 4. | RP23-130D7 | B2 | 62331567 - 62541404 | Mapping of transgene on chromosome 11 |
| 5. | RP23-125C16 | B3 | 63535367 - 63764276 | Mapping of transgene on chromosome 11 |
| 6. | RP23-355B12 | B3 | 64887956 - 65096168 | Mapping of transgene on chromosome 11 |
| 7. | RP23-298L23 | B3 | 65215576 - 65399933 | Mapping of transgene on chromosome 11 |
| 8. | RP23-287H9 | B3 | 66064135 - 66268441 | Screening of transgene on chromosome 11 |
